# A Comparative Study of Osteogenic Differentiation Human Induced Pluripotent Stem Cells and Adipose Tissue Derived Mesenchymal Stem Cells

**Published:** 2014-10-04

**Authors:** Abdolreza Ardeshirylajimi, Masoud Soleimani, Saman Hosseinkhani, Kazem Parivar, Parichehr Yaghmaei

**Affiliations:** 1 Department of Biology, Science and Research Branch, Islamic Azad University, Tehran, Iran; 2 Department of Hematology, Faculty of Medical Science, Tarbiat Modares University, Tehran, Iran; 3 Department of Biochemistry, Faculty of Biological Sciences, Tarbiat Modares University, Tehran, Iran

**Keywords:** Osteogenic, Tissue Engineering, Mesenchymal Stem Cells, Flow Cytometry, Gene Expression

## Abstract

**Objective:**

Human induced pluripotent stem cells (iPSCs) have been shown to have promising capacity for stem cell therapy and tissue engineering applications. Therefore, it is essential to compare the ability of these cells with the commonly used mesenchymal stem cells (MSC) for bone tissue engineering *in vitro*.

**Materials and Methods:**

In this experimental study, the biological behavior and osteo- genic capacity of the iPSCs were compared with MSCs isolated from human adipose tissue (AT-MSCs) using 3-(4,5-di-methylthiazol-2-yl)-2,5-diphenyltetrazolium bromide (MTT) assay, Alizarin red staining, alkaline phosphatase (ALP) activity measurements, calcium content assay and common osteogenic-related genes. Data were reported as the mean ± SD. One-way analysis of variance (ANOVA) was used to compare the results. A p value of less than 0.05 was considered statistically significant.

**Results:**

There was a significant difference between the rate of proliferation of the two types of stem cells; iPSCs showed increased proliferation compared to AT-MSCs. During osteogenic differentiation, ALP activity and mineralization were demonstrated to be significantly higher in iPSCs. Although AT-MSCs expressed higher levels of Runx2, iPSCs expressed higher levels of osteonection and osteocalcin during differentiation.

**Conclusion:**

iPSCs showed a higher capacity for osteogenic differentiation and hold promising potential for bone tissue engineering and cell therapy applications.

## Introduction

Since, bone turnover is a long and complicated process, it is essential to treat bone defects to improve regeneration and reconstruction (1). Bone tissue engineering is a new and promising treatment for bone injuries caused by infections, tumors, trauma and abnormal skeletal developments (2-4). Stem cells are noted for their potential for self-renewal and differentiation into various lineages with specific functions. In particular, their potential to give rise to mature and functional osteoblast- like cells *in vitro* and *in vivo* has attracted many scientists interested in their application as the live part of bone implants (5-7).

Mesenchymal stem cells (MSCs) are a commonly used source in regenerative medicine and tissue engineering. They have unusual and useful properties such as: the potential for intense regeneration, immunosuppressive features, and a high level of plasticity. There are different sources for deriving MSCs, like bone marrow and adipose tissue. For many years, bone marrow derived MSCs (BMMSCs) were the unique source of autologous stem cells for tissue engineering, but nowadays it has been suggested that they be replaced by adipose tissue derived MSCs (AT-MSCs) (8-10). The main reason is the increased simplicity of harvesting fat compared to bone marrow aspiration. In addition, the yield of stem cells from adipose tissue is higher than that from bone marrow (11, 12). It has been shown that the typical MSC yield for bone marrow is between 1 in 50,000 and 1 in 1 million in a skeletally mature adult and for adipose tissue is between 1 in 30 and 1 in 1,000 (11, 12). Other reasons are the low level of cell morbidity during the extraction process and the simple equipment and conditions required for the isolation and growth of AT-MSCs (13, 14).

Recently, Takahashi et al. generated induced pluripotent stem cells (iPSCs) via the transduction of murine and human fibroblasts using only four transcription factors; Oct4, Sox2, c-Myc, and Klf4 (15, 16). These cells were very similar to embryonic stem cells (ESCs) in characteristics such as morphology, gene expression, proliferation, epigenetic status of pluripotent cellspecific genes and telomerase activity. Today, advances in iPSC research have modified the prospect for tissue engineering and regenerative medicine. Several studies have reported the potential application of iPSCs in the treatment of various diseases (17, 18).

Many studies have compared the proliferation and differentiation characteristics of human ATMSCs with those of BM-MSCs (19-21), and a few studies have compared human MSCs with unrestricted somatic stem cells (USSC) isolated from umbilical cord blood (22). There have been many reports on the potential of AT-MSCs (23) and recently, we ourselves demonstrated the high proliferation and osteogenic differentiation potential of human iPSCs (24). However, as yet, there are no analyses which compare the osteogenic differentiation potential of human iPSCs with AT-MSCs. Therefore, in the present study, AT-MSCs were isolated, characterized and compared with human iPSCs based on their biological behavior and their capacity for differentiation into osteoblastic lineage.

## Materials and Methods

### Cell culture

#### Isolation and expansion of adipose tissue derived mesenchymal stem cells

In this experimental study, for isolation of ATMSCs, adipose tissue samples were collected during liposuction operations from five donors (mean age 40 ± 5, Erfan Hospital, Tehran, Iran) after informed consent according to guidelines of the Medical Ethics Committee, Ministry of Health I. R. Iran. These samples were then were treated with 0.2% collagenase II under intermittent shaking at 37˚C for 30 minutes. After centrifugation (1200 RPM for 15 minutes), the supernatant was discarded and the cell pellet was treated with RBC lysis buffer (Dako, Glostrup, Denmark) at room temperature (RT) for 5 minutes. Samples were then centrifuged at 1200 RPM for 5 minutes and the cell pellet was resuspended in a 75 cm^2^ culture flask (Nunk) in Dulbecco’s Modified Eagle’s Medium (DMEM, Invitrogen Co., Carlsbad, CA, USA) with 10% Fetal Bovine Serum (FBS, Invitrogen Co., Carlsbad, CA, USA) and incubated in 95% air and 5% CO_2_ at 37˚C. After reaching confluence (about 80-85%) over ten days the cells were detached using trypsin (2 minutes in 37˚C, 5% CO_2_) and replated.

#### Expansion of induced pluripotent stem cells

Human Fibroblast iPSC lines were obtained from the cell bank (Stem Cells Technology Research Center (Tehran, Iran) (20). These cells were expanded according to the protocol reported in a recent study (24). In brief, the cells were maintained on feeder layers of SNL76/7 cells treated with mitomycin-C (Invitrogen Co., Carlsbad, CA, USA). Human iPSC medium consisted of DMEM/F12 culture medium supplemented with 15% knockout serum replacement (KSR) (Invitrogen Co., Carlsbad, CA, USA), 0.1 mmol/L nonessential amino acids, 1 mmol/L L-glutamine (all from Invitrogen Co., Carlsbad, CA, USA), 0.1 mmol/L b-mercaptoethanol (Sigma, Munich, Germany), penicillin/ streptomycin (Sigma, Munich, Germany) and 4 ng/mL of human fibroblast growth factor 2 (Invitrogen Co., Carlsbad, CA, USA), and about 60% of the medium was replaced every day. Every four to five days human iPSC colonies were detached with 0.1% Collagenase IV, and replated onto inactivated SNL76/7 for expansion.

#### MTT assay

AT-MSCs at passage 2 (P2) and human iPSCs were seeded with an initial cell density of 5000 cells/well in 24-well tissue culture polystyrene (TCPS) plates and were cultured for five days. The proliferation of these cells was evaluated via MTT assay. Thus on each day, 50 μL of MTT solution (5 mg/mL in DMEM) was added to each well (n=4), to evaluate the conversion of MTT to formazan crystals by the mitochondrial dehydrogenases of the living cells. The plate was incubated at 37˚C, 5% CO_2_. After 3.5 hour incubation the supernatant was removed to examine the dissolution of the dark-blue intracellular formazan, and 250 μL dimethyl sulfoxide (DMSO) as an appropriate solvent was added. The optical density was read at a wavelength of 570 nm in a micro-plate reader (ELx-800, BIOTEK instruments, Winooski, VT, USA).

For osteo-lineage differentiation, the cells were cultured in osteogenesis medium containing basal medium (DMEM + FBS 10%) supplemented with 10 nM dexamethasone, 0.2 mM ascorbic acid 2-phosphate, and 10 mM β-glycerophosphate (all from sigma, Munich, Germany). The medium was changed every 2 days. Finally, at day 21 of culture in inductive medium the cells were stained with alizarin red S to assess mineralization.

#### Flow cytometry

After two weeks of culture, the expression of surface markers was evaluated using monoclonal antibodies including Fluorescent isothiocyanate (FITC)-conjugated mouse anti-human CD45 (leukocyte common antigen), Phycoerythrin (PE)-conjugated CD105 (Endoglin or SH2) CD34, and CD90. The cells were detached with trypsin/EDTA and incubated with the specific antibodies or isotype control antibodies (FITC-or PE-labeled antibodies were included in each experiment) in 100 μL of 3% bovine serum albumin in PBS for 1 hour at 4˚C. The cells were then fixed with 1% paraformaldehyde and analyzed with a Coulter Epics-XL flow cytometer (Beckman Coulter, Fullerton, CA, USA) and Win MDI 2.8 software (Scripps Institute, La Jolla, CA, USA).

#### Alkaline phosphatase activity and calcium content assay

ALP activity measurement was performed by total protein extraction of cells using 200 μL radio immune precipitation assay (RIPA) lysis buffer. Then, the lysate was centrifuged at 15000 g at 4˚C for 15 minutes, after the collection of supernatant, ALP activity was measured using an ALP assay kit (Parsazmun, Tehran, Iran) according to the manufacturer’s protocol. Activity of the enzyme (IU/L) was normalized against total protein (mg). Mineralization, as a late marker in the osteogenic differentiation of stem cells, was also quantified. During osteogenic differentiation, the amount of calcium minerals deposited on stem cells was measured using the cresolphthalein complexone method with a calcium content assay kit (Parsazmun, Tehran, Iran). Calcium extraction was performed by 0.6 N HCL (Merck, Darmstadt, Germany). After the addition of the reagent to calcium solutions, optical density (OD) was measured at 570 nm. Calcium content was obtained from the standard curve of OD versus a serial dilution of calcium concentrations.

#### Real-time reverse transcriptase-polymerase chain reaction (RT-PCR)

The difference between the mRNA levels of important bone-related genes in stem cells was analyzed using real-time RT-PCR. Total RNA was extracted and random hexamer primed cDNA synthesis was carried out using Revert Aid first strand cDNA synthesis kit (Fermentas, Burlington, Canada). The cDNAs were used for 40 cycle PCR in a Rotor-gene Q real-time analyzer (Corbett, Sydney, Australia). Real-time RT-PCR was performed using Maxima™ SYBR Green/ROX qPCR Master Mix (Fermentas) followed by melting curve analysis to confirm PCR specificity. Each reaction was repeated twice and threshold cycle average was used for data analysis by Rotor-gene Q software (Corbett, Sydney, Australia). Genes and related specific primers are illustrated in table 1. Relative expression was quantified using ΔΔCt method. Target genes were normalized against HPRT and calibrated to iPSCs.

**Table 1:** Primers used in real-time RT-PCR


Gene	Primer sequence	Product length (base pairs)

**HPRT1**	CCTGGCGTCGTGATTAGTG TCAGTCCTGTCCATAATTAGTCC	125
**Runx2**	GCCTTCAAGGTGGTAGCCC CGTTACCCGCCATGACAGTA	67
**Osteonectin**	AGGTATCTGTGGGAGCTAATC ATTGCTGCACACCTTCTC	121
**Osteocalcin**	GCAAAGGTGCAGCCTTTGTG GGCTCCCAGCCATTGATACAG	224
**Oct-4**	GTTCTATTTG GGAAGGTATTC CAGCTTACACATGTTCTTGAA	83
**Sox-2**	GGACTGAGAGAAAGAAGAGGAG GAAAATCAGGCGAAGAATAAT	196
**Nanog**	GCTAAGGACAACATTGATAGAAG CTTCATCACCAATTCGTACTTG	128


HPRT1; Hypoxanthine phosphoribosyltransferase 1, Runx2; Runt-related transcription factor 2, Oct4; Octamer-binding transcription factor 4 and Sox2; SRY (sex determining region Y)-box 2.

#### Statistical analysis

All experiments were conducted at least for three times. Data were reported as the mean ± SD. One-way analysis of variance (ANOVA) was used to compare the results. A p value of less than 0.05 was considered statistically significant. All statistical analyses were conducted with SPSS software, version 11.0 (SPSS, Chicago, IL, USA).

## Results

### Characterization of adipose tissue derived mesenchymal stem cells

**Fig 1:**
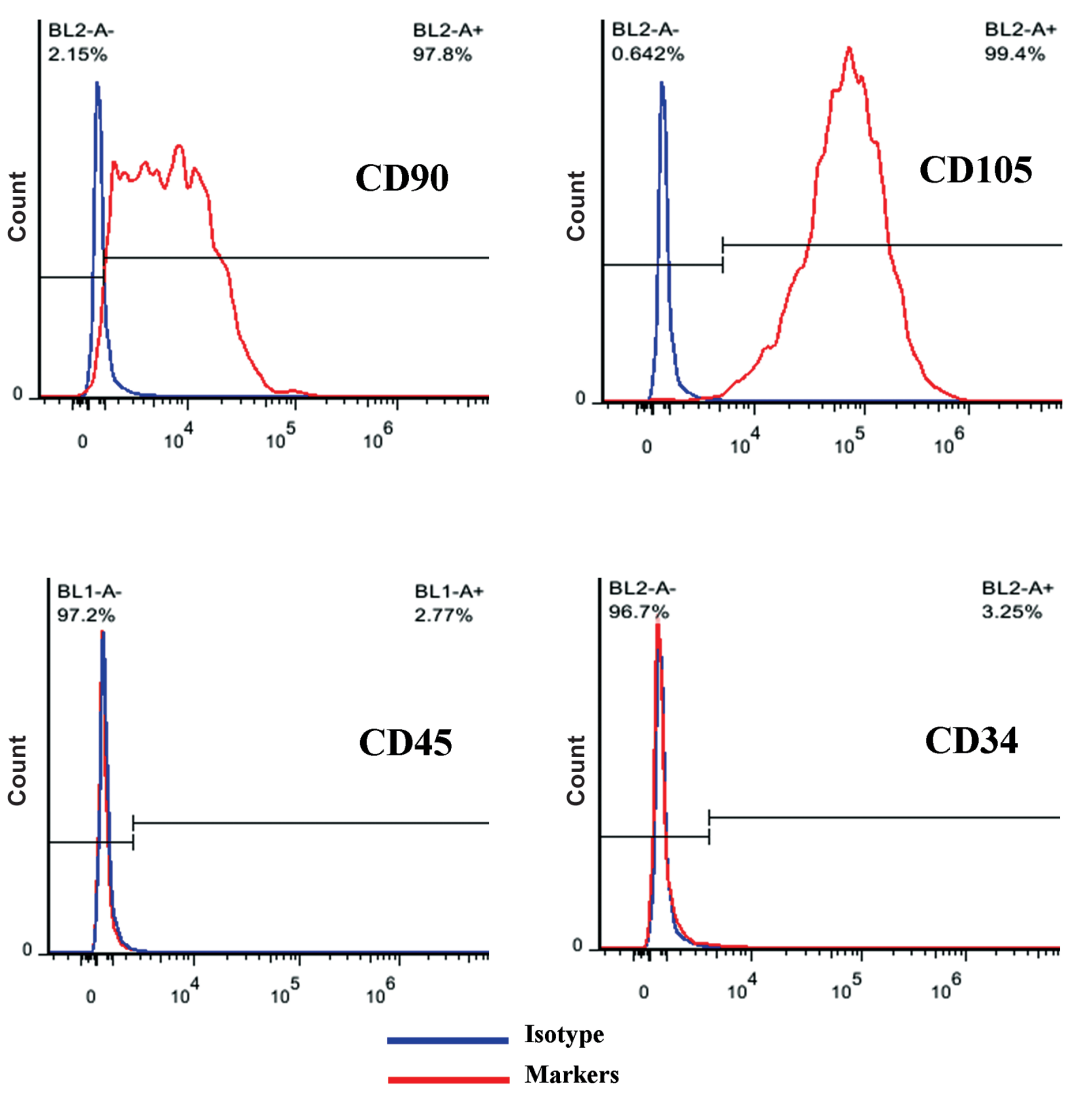
Flow cytometry analysis of AT-MSCs.

**Fig 2:**
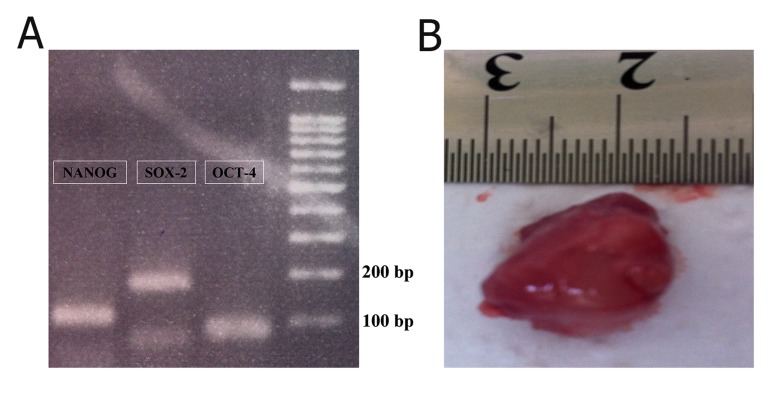
Pluripotency of induced pluripotent stem cells established using the expression of Oct-4, Sox-2 and Nanog(A) and the explanted teratoma (B).

**Fig 3:**
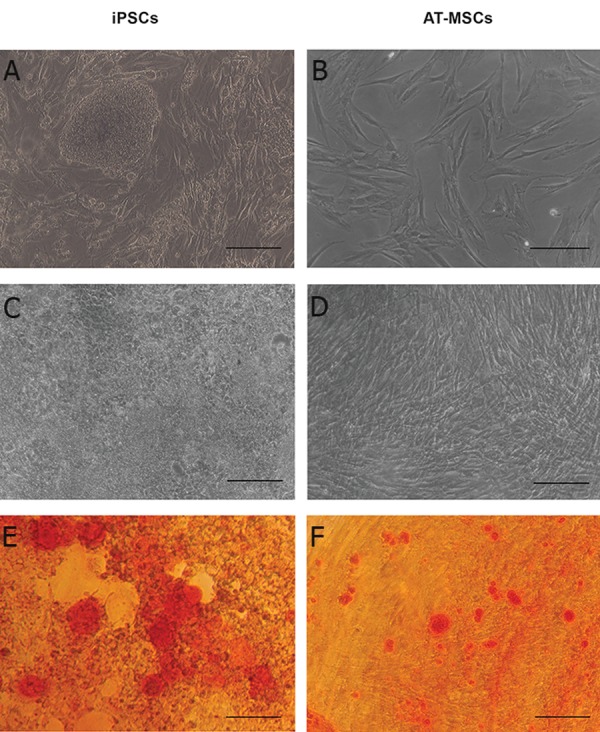
The morphology of stem cells under basal medium [iPSCs (A) and AT-MSCs (B)]; after 21 days culture in osteogenic induction medium [iPSCs (C) and AT-MSCs (D)]; and alizarin red staining of stem cells after 21 days culture in osteogenic induction medium [iPSCs (E) and AT-MSCs (F)], scale bars (100 μm).

**Fig 4:**
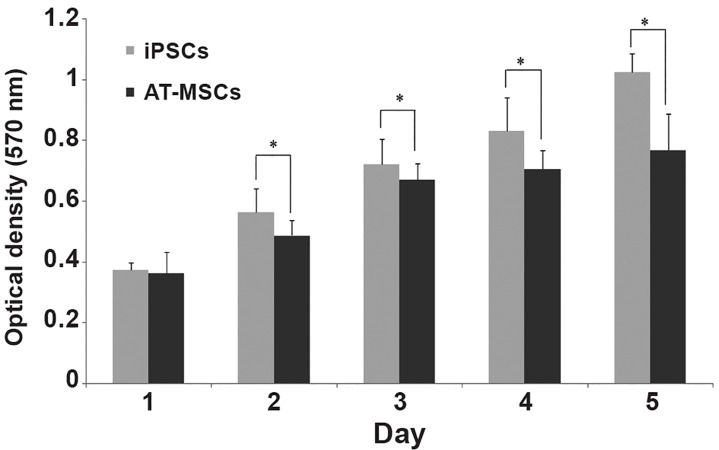
Proliferation of induced pluripotent stem cells (iPSCs) and adipose tissue derived mesenchymal stem cells (AT-MSCs) on tissue culture polystyrene (TCPS) over a 5 day culture period (asterisk shows significant difference, p<0.05).

Isolated stem cells from adipose tissue were characterized based on their surface markers. AT-MSCs were negative for CD45 and CD34 and were positive for CD90 and CD105 (Fig 1). iPSCs were also characterized using RT-PCR for pluripotency genes and teratoma formation capability (Fig 2). Morphology of human iPSC colonies seeded on SNL76/7 feeder layers were similar to human ES morphology (Fig 3A) and AT-MSCs also showed fibroblast-like and spindle- shaped morphology when cultured at lowdensity (Fig 3B). In osteogenic medium (Fig 3C-D), the biomineralization and secreted extracellular matrix were clearly observed on both types of stem cell and were visualized by Alizarin red staining (Fig 3E-F). Calcium depositions in the two types of stem cell represented a uniform pattern. However, different amounts of mineralization were observed in various fields of AT-MSCs and iPSCs monolayer. The proliferation rate of stem cells was also significantly different (Fig 4). According to the results, a higher rate of proliferation was observed in iPSCs compared to AT-MSCs.

### Alkaline phosphatase activity and mineralization of stem cells

**Fig 5:**
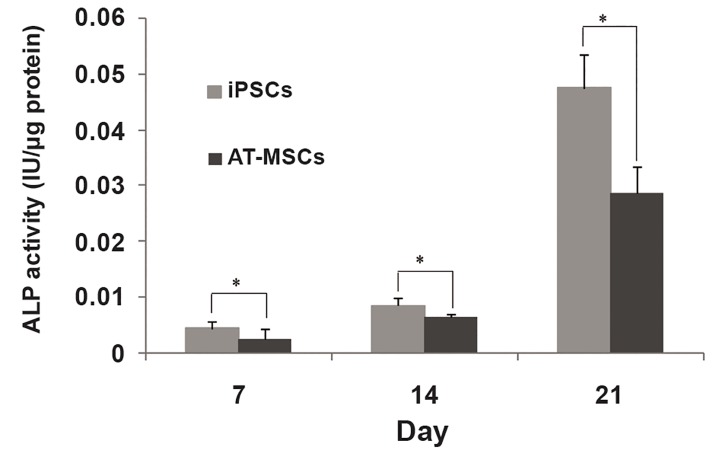
ALP activity of stem cells during osteogenic differentiation [asterisk shows significant difference between adipose tissue derived mesenchymal stem cells (AT-MSCs) and induced pluripotent stem cells (iPSCs) on each day, p<0.05].

**Fig 6:**
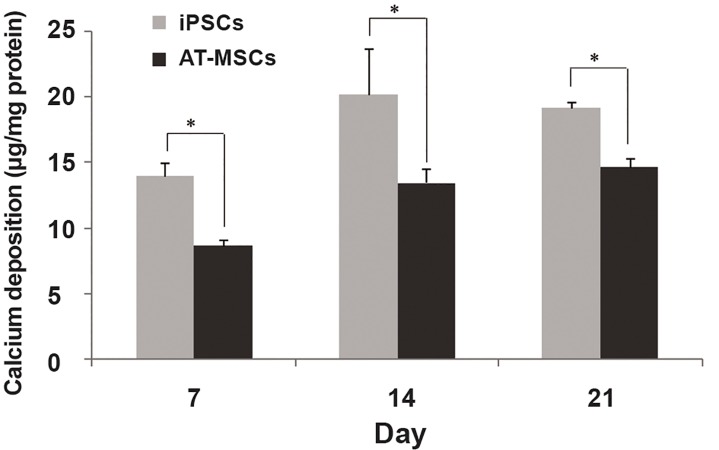
Calcium content of stem cells during osteogenic differentiation [asterisk shows significant difference between AT-MSsC and iPSCs on each day at p<0.05].

ALP activity, as a marker of osteogenesis in stem cells, was measured during induction of osteogenesis (Fig 5). ALP activity in all stem cells showed a similar pattern during osteogenic differentiation. AT-MSCs showed significantly higher ALP activity than iPSCs at two time points (14 and 21 days). Furthermore, higher ALP activity was measured in iPSCs compared to AT-MSCs on day 7, however, the difference was not significant (Fig 6). The calcium content of both stem cells displayed a similar pattern, including a peak during osteogenic differentiation. iPSCs demonstrated a higher level of calcium deposition than AT-MSCs at all time points, with a peak on day 14. Furthermore, significantly higher calcium deposition was observed in iPSCs compared to AT-MSCs at all time points.

### Gene expression analysis

**Fig 7:**
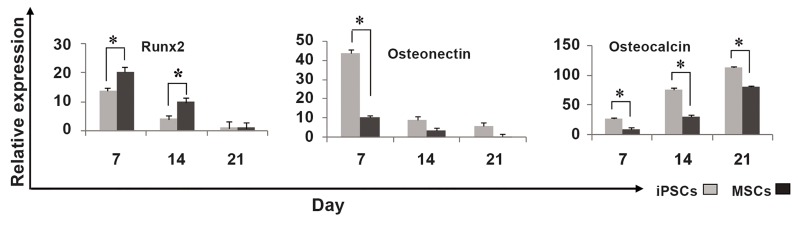
Relative expression of Osteocalcin, Runx2 and Osteonectin in stem cells on days 7, 14 and 21 during osteogenic differentiation (asterisk shows significant difference between two groups on each day at p<0.05).

The relative expression of three important bone-related genes was investigated during osteogenic differentiation of the two stem cells (Fig 7). A decreasing trend in the expression of Runt-related transcription factor 2 (Runx2) was observed in iPSCs and AT-MSCs during osteogenic differentiation. This gene was expressed at a higher level on days 7 and 14 in iPSCs compared to AT-MSCs. The highest amount of osteonectin was observed in iPSCs and AT-MSCs on day 7. In both types of stem cell, osteonectin expression was down-regulated on days 14 and 21. In both types of stem cell, the expression of osteocalcin increased similarly during culture. The expression of osteocalcin during osteogenic differentiation increased significantly in iPSCs compared to AT-MSCs.

## Discussion

Currently the use of stem cells to promote bone healing is being progressed and developed because of the unique characteristics of these cells, such as extensive proliferation, differentiation and growth factor secretion at the site of osseous defects (25- 27). Several studies have shown that CD90 (thymocyte differentiation antigen 1) and CD105 (known as endoglin) are surface markers that must be expressed on the surface of the so-called MSCs (28). These cells also must lack expression of CD45 and CD34 which showed their non-hematopoietic origin (29-31). Flow cytometric data confirmed that the isolated cells from adipose tissue are mesenchymal. In the present study, both stem cell types showed similar patterns of ALP activity during differentiation into osteoblasts. ALP has a critical role in osteogenesis due to its role in the cleavage of calcium phosphate groups and enhancment of mineralization of the calcium phosphate cements which are a hallmark of the final osteogenic differentiation of stem cells *in vitro* (32-34). Our ALP activity data showed that iPSCs have superior osteogenic capacity compared with AT-MSCs at all time points. In addition, mineralization data confirmed the patterns of ALP activity in both stem cells. Matrix mineralization is a result of continuous mineral deposition due to ALP activity, crystal nucleation and precipitation during osteogenic differentiation (35). The significant difference observed between the level of mineralization in iPSC compared to AT-MSCs suggests that the movement of iPSCs toward osteogenic lineage is faster and higher yield. It seems that this effect could be a result of the high proliferation rate of iPSCs, as findings from several studies have demonstrated that one of the most important items in osteogenic differentiation is confluency of stem cells (36, 37). Alizarin red staining showed no significant difference between the two stem cell types. This result can be explained by the heterogeneous initial cell seeding and differential rate of cell attachment.

In this study, three important bone-related genes were selected and their expression was evaluated for better comparison and understanding of the osteogenic differentiation behavior of iPSCs and ATMSCs. Runx2 is an early osteogenic differentiation gene marker. Up-regulation and significantly higher levels of Runx2 were observed on days 7 and 14 in AT-MSCs compared to iPSCs. The superior mineralization of iPSCs can be explained by up-regulation of the osteonectin gene due to the important role of osteonectin expression in initial crystal growth on stem cells during osteogenic differentiation. This can also explain the higher expression of osteocalcin in iPSCs compared to AT-MSCs. Both stem cell types showed a similar pattern of expression of osteocalcin, but higher expression of this gene was observed in iPSCs compared to AT-MSCs. Therefore, high expression of osteocalcin and down-regulation of Runx2 and osteonectin as critical osteogenic genes in both types of stem cell can be a good predictor of their capacity for differentiation into osteogenic lineage. In addition, iPSCs showed better results than ATMSCs in relation to osteogenic gene expression, which confirmed the higher osteogenic differentiation potential in iPSCs compared to AT-MSCs.

## Conclusion

Taking all the results together, iPSCs showed a superior capacity for osteogenic differentiation than AT-MSCs. Although the capacity for osteogenic differentiation is an important factor when selecting the cell source for bone cell-based therapies, the rate of proliferation and senescence-associated characteristics should also be considered in the isolation, expansion and selection process.
